# A Highly Conserved Glycine in a Hotspot for Neurological Disease Mutations in Na^+^,K^+^-ATPase Is Critical to Na^+^ and K^+^ Occlusion

**DOI:** 10.3390/biom16040601

**Published:** 2026-04-17

**Authors:** Mads S. Toustrup-Jensen, Rikke Holm, Jens Peter Andersen, Bente Vilsen

**Affiliations:** Department of Biomedicine, Aarhus University, DK-8000 Aarhus, Denmark; rhj@biomed.au.dk (R.H.); jpa@biomed.au.dk (J.P.A.)

**Keywords:** Na^+^,K^+^-pump, neurological disease, P-type ATPase, mutagenesis, glycine, phosphorylation, oligomycin, Na^+^ site, K^+^ site, transient kinetics

## Abstract

Na^+^,K^+^-ATPase possesses a highly conserved glycine (G358 in the α3 isoform) that—together with a nearby isoleucine (I363 in α3)—is targeted by mutations causing some of the most severe neurological phenotypes of the clinical spectrum of α3-Na^+^,K^+^-ATPase mutations. The disease mutations α3-G358V and α3-I363N affect Na^+^ and K^+^ transport to an extent incompatible with cell growth. However, alanine replacement of the corresponding glycine G363 in the α1 isoform is compatible with cell growth, allowing the effects on Na^+^,K^+^-ATPase function to be addressed using enzymatic assays on plasma membranes isolated from transfected cells. Occlusion of Na^+^ appears to be defective in mutant G363A, resulting in a reduced rate of phosphorylation from ATP. Furthermore, the mutation displaces the major conformational equilibrium of Na^+^,K^+^-ATPase such that the K^+^-occluded state is destabilized and occluded K^+^ is released faster, thereby leading to accumulation of a non-productive state without bound Na^+^ or K^+^. The critical function of the glycine can be ascribed to a strategic location at the bending point between an α helix and a β strand, where it connects the catalytic ATP hydrolysis site in the cytoplasmic P domain with the ion-binding region in the membrane and coordinates important intramolecular domain movements during the Na^+^,K^+^-ATPase transport cycle.

## 1. Introduction

**Figure 1:**
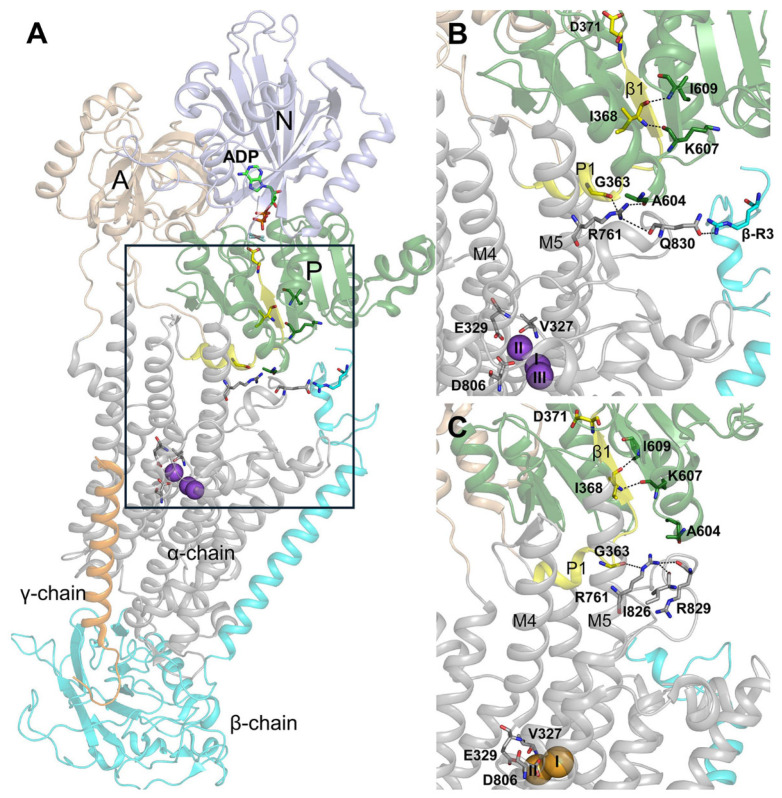
Structural features relevant to the present study. (**A**) Overall structure of Na^+^,K^+^-ATPase in [Na_3_]E_1_P form, PDB accession code 3WGV. (**B**) Zoomed-in view of the boxed region. (**C**) Zoomed-in view of the corresponding region of the K^+^-occluded [K_2_]E_2_ structure (PDB accession code 7Y45). The cytoplasmic side is upward and the extracellular side downward. The P-, N-, and A domains of the cytoplasmic headpiece of the α subunit (α chain) are colored green, light blue, and sandy, respectively, except for the P1 helix containing G363 and the β1 strand containing I368 in focus in the present study being highlighted by yellow, though belonging to the P domain. The transmembrane part of the α chain, consisting mainly of the ten helices M1–M10 (M4 and M5 being labeled), is colored gray, the β chain cyan, and the γ chain (FXYD subunit) pale orange. ADP in the catalytic site, and residues relevant to the present study are highlighted in stick form, with oxygen atoms red and nitrogen atoms blue. Residue numbering is as per the rat α1 isoform. The P1 helix and the β1 strand connect the catalytic site (represented by the phosphorylated aspartate D371) with the M4 helix, whose movement is crucial to the translocation of Na^+^ and K^+^. Purple spheres labeled I, II, and III represent the Na^+^ ions occluded in [Na_3_]E_1_P. Bronze-colored spheres labeled I and II represent the two K^+^ ions occluded in [K_2_]E_2_. Numbering of the ions is as per accepted nomenclature [6]. Two of the ion-binding residues of M4, E329, and V327, and one of the ion-binding residues belonging to M6, D806, are highlighted in stick form. The dotted lines indicate hydrogen bonds belonging to the networks involving G363 and I368.

**Scheme 1:**
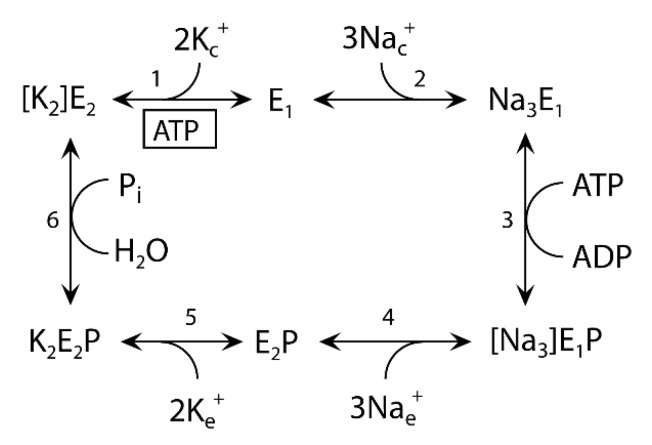
Na^+^,K^+^-ATPase reaction cycle [2,3,4]. The partial reactions are numbered 1–6, the forward-running mode being clockwise. E represents the Na^+^,K^+^-ATPase enzyme. There are four main conformational states, the unphosphorylated E_1_ and E_2_ states and the phosphorylated E_1_P and E_2_P states (P symbolizing phosphorylation of the invariant aspartate in the DKTGT P-type ATPase signature motif). Free Na^+^ and K^+^ ions in the medium are labeled c and e for cytoplasmic and extracellular side, respectively. Occluded Na^+^ and K^+^ ions (i.e., bound in the membrane sector of the protein in a closed cavity without access to medium on either side of the membrane) are shown in brackets, whereas the exchangeable Na^+^ and K^+^ ions bound at open sites are shown without brackets. E_1_ binds three Na^+^ with high affinity or two K^+^ with low affinity at open cytoplasmic-facing sites. K^+^ binding converts E_1_ to the K^+^-occluded [K_2_]E_2_ state (so-called direct route of K^+^ occlusion). Phosphorylation of E_1_ from ATP can only occur with three Na^+^ bound, leading to the [Na_3_]E_1_P intermediate containing Na^+^ in an occluded state. This phosphoenzyme intermediate is “ADP-sensitive,” being able to donate the phosphoryl group back to ADP. In the forward-running mode of the cycle, [Na_3_]E_1_P is converted to the “ADP-insensitive” and “K^+^-sensitive” E_2_P intermediate with the extracellular release of Na^+^. This is followed by binding of two K^+^ with high affinity at the extracellular-facing sites of E_2_P, which stimulates the dephosphorylation, leading to [K_2_]E_2_. Boxed ATP indicates ATP bound in a non-phosphorylating allosteric mode, enhancing the rate of K^+^ deocclusion and accompanying E_2_–E_1_ conformational change.

Na^+^,K^+^-ATPase is a transmembrane enzyme fundamental to all mammalian cells, as it utilizes the energy stored in ATP to transport Na^+^ out of the cell and K^+^ into the cell, thereby creating indispensable electrochemical gradients for these ions across the cell membrane [1,2,3,4]. It belongs to the family of transport ATPases named P-type ATPases due to the formation of a phosphorylated enzyme intermediate during the transport cycle in which the γ-phosphate of ATP is transferred to a conserved aspartate residue located in the P domain within the cytoplasmic headpiece of the catalytic α subunit. The α subunit contains the binding sites for ATP and the transported ions (the latter located in the transmembrane part). In addition, the Na^+^,K^+^-ATPase protein consists of a β subunit that functions as a chaperone facilitating the folding and plasma membrane insertion of the α subunit. A regulatory subunit belonging to the FXYD protein family is present in most cells (the FXYD2 protein expressed in kidney is denoted γ subunit; see Figure 1A). Detailed structural information from X-ray crystallography and cryo-EM exists for various conformational states in the transport cycle, including Na^+^- and K^+^-bound forms [4,5,6,7,8]—cf Figure 1 and Scheme 1. Four different genes encode the α subunit, resulting in tissue-specific and developmentally regulated expression of four highly homologous isoforms: α1–α4 [9]. Most tissues express the α1 isoform, whereas the α2 and α3 isoforms are expressed mainly in the nervous system and muscle, α2 in glial cells and α3 in neurons. Both these cell types also express α1 [10]. Neurological disorders have been found associated with mutation of any of these three isoforms [11,12,13,14,15,16,17,18,19,20,21,22,23,24,25,26].

As in other P-type ATPases, the cytoplasmic headpiece of the α subunit of Na^+^,K^+^-ATPase is divided into three separate domains: nucleotide-binding (“N”), phosphorylation (“P”) and actuator (“A”), see Figure 1. The cytoplasmic headpiece is linked to ten transmembrane helices (M1–M10). The amino-acid residues with oxygen-containing side chains being directly involved in the binding and occlusion of Na^+^ and K^+^ during the transport process are situated in M4, M5, M6, and M8 [4,5,6,7,8]. Most P-type ATPases, including Na^+^,K^+^-ATPases, H^+^,K^+^-ATPases, Ca^2+^-ATPases, as well as P4- and P5-ATPases, contain a highly conserved glycine (G363 in Figure 1) strategically placed at the bending point between the first α helix of the P domain (“P1 helix”) and the following β strand (“β1”), both colored yellow in Figure 1, leading directly to the catalytic site containing the phosphorylated aspartate in the P-type ATPase signature motif DKTGT (D371 in Figure 1). This glycine residue is furthermore connected through the P1 helix to transmembrane segment M4, which plays a crucial role in the binding and translocation of Na^+^ and K^+^ [6,7]. The backbone carbonyl group of the glycine forms a hydrogen bond with a centrally located arginine in transmembrane helix M5 (R761 in Figure 1). The glycine and the arginine, together with an isoleucine (I368 in Figure 1) five residues downstream of the glycine and only three residues from the phosphorylated aspartate, form a hotspot of residues targeted by neurological disease mutations in the α3 isoform of Na^+^,K^+^-ATPase [24,25]. The pathogenic glycine and isoleucine mutations (human α3-G358V and α3-I363N) are associated with some of the most severe neurological phenotypes of the clinical spectrum reported for α3 mutations, the first with catastrophic early-life epilepsy and the other with epilepsy and life-threatening recurrent apnea leading to a severely impaired neurodevelopmental outcome [24]. In the present study, these disease-causing mutations of human α3, as well as their rat α1 equivalents G363V and I368N, were found to affect Na^+^,K^+^-ATPase function to an extent incompatible with cell growth, thus precluding membrane isolation and enzymatic studies. However, the G363A mutation of the corresponding glycine in rat α1 was compatible with cell growth, allowing its functional effects on the Na^+^,K^+^-ATPase to be addressed and characterized in detail using enzymatic assays on plasma membranes isolated from transfected cells. The results demonstrate a highly critical role of the glycine in coordinating intramolecular domain movements involved in occlusion of Na^+^ and K^+^ during the Na^+^,K^+^-ATPase transport cycle.

## 2. Materials and Methods

### 2.1. Mutagenesis and Expression

The cDNA encoding either the ouabain-insensitive rat α1 isoform of Na^+^,K^+^-ATPase or the human α3 isoform previously made ouabain-resistant by mutations Q108R and N119D [27] was subjected to PCR-based mutagenesis to introduce the desired point mutations, which were verified by full-length sequencing. The Ca^2+^-phosphate precipitation method [28] was applied to transfect mutant or wild-type cDNA into COS-1 cells [29], which had been acquired from the American Type Culture Collection (ATCC, CRL-1650; Manassas, VA, USA). The cells were treated with 10% glycerol to enhance DNA uptake and were grown in the presence of 5 µM ouabain to inhibit the endogenous COS-1 cell Na^+^,K^+^-ATPase [30]. Under such ouabain selection pressure, only cells expressing an active exogenous ouabain-insensitive Na^+^,K^+^-ATPase are viable, allowing stable cell lines to be isolated [27,30]. The expression level of the exogenous enzyme increases with time under ouabain selection pressure, while the level of the inhibited endogenous enzyme being replaced decreases [31]. As described in Results, viable cells were obtained with the G363A mutant. The cells were grown until the expression level was fully optimized, which took considerably longer than for the wild type, because the growth of the G363A cells was slow as a consequence of the low catalytic turnover rate of the mutant (see further in Section 3).

### 2.2. Preparation of Plasma Membranes and ATPase Activity Measurements

Plasma membrane vesicles from stable COS-1 cell lines expressing the active G363A mutant or wild-type Na^+^,K^+^-ATPase were harvested using differential centrifugation. To ensure access of ATP and ions to the Na^+^,K^+^-ATPase from both sides of the membrane, the vesicles were permeabilized by incubation with deoxycholate or alamethicin as previously described [30]. The Na^+^,K^+^-ATPase activity of the leaky membrane preparation was determined at 37 °C using the colorimetric procedure of Baginski [32] to follow the liberation of P_i_ from ATP for 15 min (i.e., within the linear time range) in medium containing, in addition to the membranes (20 μg membrane protein/mL), 30 mM histidine (pH 7.4), 1 mM EGTA, 3 mM MgCl_2_, 10 µM ouabain (to silence the endogenous COS-1 cell Na^+^,K^+^-ATPase), and the concentrations of NaCl, KCl, and ATP described in the figure legends. To obtain the ATPase activity specifically associated with Na^+^,K^+^-ATPase, background ATPase activity ascribable to ATPases other than Na^+^,K^+^-ATPase, determined in the presence of 10 mM ouabain to inhibit all Na^+^,K^+^-ATPase activity, was subtracted.

### 2.3. Oligomycin Effect, Na^+^ Dependence, and Rapid Kinetic Measurements of Phosphorylation

Phosphorylation studies were performed on the leaky plasma membrane preparation as per previously established principles [33,34]. The effect of oligomycin on phosphorylation was examined by incubating the plasma membranes (10 µg membrane protein) at 0 °C with 2 µM [γ-^32^P]ATP (specific activity as previously described [30]) in a total volume of 100 µL medium containing 20 mM Tris (pH 7.5), 3 mM MgCl_2_, 1 mM EGTA, 10 µM ouabain (preventing phosphorylation of the endogenous COS-1 cell Na^+^,K^+^-ATPase), and 150 mM NaCl, with a variable concentration (0–75 µM) of oligomycin (a mixture of oligomycin A, B, and C isomers from Sigma-Aldrich). To ensure that the endogenous COS-1 cell Na^+^,K^+^-ATPase had bound ouabain prior to the initiation of the phosphorylation reaction with [γ-^32^P]ATP, the plasma membranes were preincubated with ouabain for 30 min at room temperature in medium containing only Tris (pH 7.5), MgCl_2_, and ouabain. To determine the apparent affinity for Na^+^, phosphorylation was carried out as described above in the presence of 25 µM oligomycin and varying NaCl concentrations (N-methyl-D-glucamine was added at concentrations keeping the ionic strength constant at 150 mM). Using vertically orientated 8 mm x 1 mm magnet bars, the reaction mixture was stirred continually during phosphorylation in an Eppendorf tube immersed in ice water. After 10 s of phosphorylation, 500 µL ice-cold quench solution (1.1 M phosphoric acid, pH 2.4, adjusted with NaOH) was added, the timing being controlled by an electronic metronome.

By use of a QFM-5 quench-flow module (Bio-Logic Sciences Instruments, Claix, France), rapid kinetic measurements of the time course of phosphorylation at 25 °C were performed as per the previously described “Protocol 1” [34]. The plasma membranes in medium containing 100 mM NaCl, 40 mM Tris (pH 7.4), 3 mM MgCl_2_, 1 mM EGTA, 10 µM ouabain (preventing phosphorylation of the endogenous COS-1 cell Na^+^,K^+^-ATPase), and 25 µM oligomycin were mixed with an equal volume of the same medium with [γ-^32^P]ATP (1, 2, 4, or 10 µM), followed by acid-quenching at various time intervals with a double volume of 2.5 M phosphoric acid, pH 2.4 (adjusted with NaOH). Reaction loop volumes (20 µL or 100 µL) and flow rate were varied to obtain reaction times between 11 and 300 milliseconds.

In all the phosphorylation experiments described above, the acid-precipitated ^32^P-labeled phosphoenzyme was subjected to SDS-polyacrylamide gel electrophoresis at pH 6.0 (the relatively low pH stabilizing the phosphoenzyme) following washing with 0.25 M phosphoric acid, pH 2.4 (adjusted with NaOH) by centrifugation. The separated ^32^P-labeled phosphoenzyme band was visualized and quantified by phosphor imaging using a cyclone storage system (PerkinElmer, Shelton, CT, USA). Background phosphorylation of proteins other than Na^+^,K^+^-ATPase was determined in medium containing 50 mM KCl instead of NaCl and subtracted. For 10 s phosphorylation experiments, the background constituted less than 20% of the total phosphorylation. For rapid kinetic measurements, the background was less than 5%.

### 2.4. K^+^ and Rb^+^ Occlusion and Time Course of Deocclusion

In this assay, the relative amount of K^+^- or Rb^+^-occluded intermediate ([K_2_]E_2_ or [Rb_2_]E_2_) was determined by taking advantage of the inability of this intermediate to phosphorylate from [γ-^32^P]ATP (phosphorylation only occurs after conversion to the Na^+^-bound E_1_ form, cf Scheme 1) [33]. The leaky plasma membranes (10 µg for each data point) were preincubated for 10 min in medium containing 20 µM ouabain (to silence the endogenous COS-1 cell Na^+^,K^+^-ATPase), 3 mM MgCl_2_, and 20 mM Tris (pH 7.5), followed by addition of KCl or RbCl at final concentrations of 1 mM and 8 mM, respectively, and incubation at room temperature for 1 h to form the K^+^- or Rb^+^-occluded [K_2_/Rb_2_]E_2_ intermediate. One minute before initiation of deocclusion, oligomycin (190 µM) was added to promote Na^+^ binding and phosphorylation from [γ-^32^P]ATP after release of K^+^ or Rb^+^. Samples, each consisting of 40 µL, were cooled to 10 °C in a thermostatically controlled water bath, followed by dilution in 360 µL of a phosphorylation medium at the same temperature, producing final concentrations of 1 µM [γ-^32^P]ATP, 100 mM NaCl, 1 mM MgCl_2_, 1 mM EGTA, 20 mM Tris (pH 7.4), 19 µM oligomycin, and 10 µM ouabain. The reaction mixture was stirred continually, as described in Section 2.3. To monitor the time course of deocclusion of K^+^ or Rb^+^ by the phosphorylation level, acid-quenching with 1.0 mL 1.5 M phosphoric acid (pH 2.4, adjusted with NaOH) was performed at the indicated time intervals after the dilution. Washing and processing of the acid-precipitated ^32^P-labeled phosphoenzyme was carried out as described above for other phosphorylation experiments in Section 2.3. The phosphorylation level corresponding to the fully deoccluded enzyme was obtained by replacing KCl or RbCl with 50 mM NaCl during the 1-h incubation. For background phosphorylation (constituting less than 10% of the maximal phosphorylation), which was subtracted in the data shown, the 100 mM NaCl in the phosphorylation medium was replaced by 50 mM KCl.

To illustrate the fraction of the enzyme trapped in the Rb^+^-occluded form during the vanadate experiment described below in Section 2.5, Rb^+^ occlusion/deocclusion was also examined under conditions resembling those of the vanadate experiment except for the absence of vanadate. The Rb^+^-occluded intermediate was formed by incubation for 30 min at 20 °C in medium containing 20 mM Tris (pH 7.5), 5 mM MgCl_2_, 1 mM EGTA, and 8 mM RbCl. Samples, each consisting of 40 µL, were cooled to 0 °C, followed by dilution in 360 µL of a phosphorylation medium at 0 °C, producing final concentrations of 50 mM NaCl, 50 mM choline chloride, 20 mM Tris (pH 7.5), 5 µM [γ-^32^P]ATP, 5 mM MgCl_2_, 1 mM EGTA, and 25 µM oligomycin. At the indicated time intervals following the dilution, acid-quenching was performed with 0.4 mL 2 M phosphoric acid (pH 2.4, adjusted with NaOH).

### 2.5. Vanadate Affinity

To examine the vanadate affinity, the leaky plasma membranes (10 µg for each data point) were incubated at 20 °C for 30 min in 40 µL medium containing 20 mM Tris (pH 7.5), 5 mM MgCl_2_, 1 mM EGTA, and various concentrations of orthovanadate, as indicated in the figure legend, without or with 8 mM RbCl (stabilizing the enzyme in the E_2_ form). To prevent dissociation of bound vanadate, the 40 µL samples were cooled to 0 °C, and the enzyme fraction with no vanadate bound was determined by measuring the amount of phosphoenzyme formed upon the addition of 360 µL ice-cold phosphorylation medium resulting in final concentrations of 50 mM NaCl, 50 mM choline chloride, 20 mM Tris (pH 7.5), 5 µM [γ-^32^P]ATP, 5 mM MgCl_2_, 1 mM EGTA, and 25 µM oligomycin. After 30 s of phosphorylation, acid-quenching was performed with 0.4 mL 2 M phosphoric acid (pH 2.4, adjusted with NaOH). Washing and processing of the acid-precipitated ^32^P-labeled phosphoenzyme proceeded as described above for other phosphorylation experiments in Section 2.3.

### 2.6. Data Analysis and Statistics

Each data point shown is the average of at least two independently performed experiments with SEM values indicated by error bars (seen only when larger than the size of the symbols). SigmaPlot software (version 10.0, SYSTAT, Palo Alto, CA, USA) was used to fit the relevant equations to the data points by non-linear regression, and the extracted parameters corresponding to the best fits are reported as ±SEM values.

The oligomycin concentration dependence of phosphorylation, the Na^+^ concentration dependence of phosphorylation, the K^+^ concentration dependence of Na^+^,K^+^-ATPase activity (wild type), and the ATP concentration dependence of Na^+^,K^+^-ATPase activity were analyzed using a Hill function (Equation (1)):

(1) $$A={(A}_{max}-A_{0})\cdot \left(\frac{{\left[L\right]}^{n}}{K_{0.5}^{n}+{\left[L\right]}^{n}}\right)+\;A_{0}$$

*A* represents the phosphorylation or activity level at the actual ligand (L) concentration, *A*_0_ is the value corresponding to a zero ligand concentration (*A*_0_ = 0 for L being Na^+^ or ATP), *A*_max_ is the extrapolated maximal phosphorylation or activity level corresponding to an infinite ligand concentration, *K*_0.5_ is the ligand concentration giving half-maximum activation (reciprocal of apparent affinity), *n* is the Hill coefficient (only deviating significantly from 1 for L being Na^+^ or K^+^, which bind at three and two sites, respectively).

Because the K^+^ concentration dependence of Na^+^,K^+^-ATPase activity of the G363A mutant is biphasic, showing inhibition at the highest K^+^ concentrations, a negative Hill function was added in this case to represent the inhibition (Equation (2)):

(2) $$A=\frac{A_{max}{\left[L\right]}^{n1}}{\left(K_{0.5A}^{n1}+{\left[L\right]}^{n1}\right)}-\frac{I_{max}{\left[L\right]}^{n2}}{\left(K_{0.5I}^{n2}+{\left[L\right]}^{n2}\right)}+\;A_{0}$$

The activation and inhibition phases are denoted *A* and *I*, respectively, while *n*1 and *n*2 are the corresponding Hill coefficients.

The time course of phosphorylation was analyzed using a mono-exponential “rise to max” function (Equation (3)):

(3) $$EP={EP}_{max}\cdot (1-e^{-k\cdot t})$$

*E*P represents the actual phosphorylation level at the given time (*t*) after initiation of the reaction, *E*P_max_ is the maximal phosphorylation level, and *k* is the rate constant.

The dependence of the initial rate of phosphorylation on the ATP concentration was analyzed using Equation (1) with *n* = 1 and *A*_0_ = 0 in the form of a linear double reciprocal plot (a classical Lineweaver–Burk plot) that provided the maximal phosphorylation rate (*A*_max_ = 1/ordinate intercept) and *K*_0.5_ for ATP (=*A*_max_∙slope).

The time course of phosphorylation starting from a mixture of E_1_ and the K^+^- or Rb^+^-occluded intermediate ([K_2_]E_2_ or [Rb_2_]E_2_) was analyzed using the biphasic time function described previously [33,34] (Equation (4)):

(4) $$EP={(EP}_{max}-E_{occluded})\cdot (1-e^{-h\cdot t})+E_{occluded}\cdot (1-e^{-k\cdot t})$$

*E*P represents the actual phosphorylation level at a given time (*t*) after initiation of deocclusion, *E*_occluded_ represents the part of the enzyme that phosphorylates slowly because of its initial presence as the occluded E_2_ form ([K_2_]E_2_ or [Rb_2_]E_2_), and *k* is the rate constant describing the slow deocclusion. *E*P_max_ − *E*_occluded_ represents the part of the enzyme initially present as the non-occluded E_1_ form that phosphorylates almost instantaneously with a rate constant *h* considerably higher than *k*. The term 1 − e^−*h*·*t*^ was set to one, because good fits were obtained for all values of *h* > 1 s^−1^ without any significant variation in the values determined for *E*_occluded_ and *k*.

The vanadate inhibition of phosphorylation was analyzed using a negative Hill function to represent the ligand-induced inhibition (Equation (5)):

(5) $$EP={EP}_{0}+{(EP}_{max}-{EP}_{0})\cdot \left(1-\frac{{\left[L\right]}^{n}}{K_{0.5}^{n}+{\left[L\right]}^{n}}\right)$$

*E*P represents the actual phosphorylation level at the given ligand concentration (L being vanadate), *E*P_0_ is the phosphorylation level corresponding to an infinite vanadate concentration (giving the maximal inhibition), *E*P_max_ is the maximal value of the phosphorylation level corresponding to a zero concentration of vanadate, *K*_0.5_ is the vanadate concentration giving half-maximum inhibition (reciprocal of apparent affinity), and *n* is the Hill coefficient (not deviating significantly from 1 for vanadate).

## 3. Results

### 3.1. Ability to Support Cell Growth

The viability and growth of mammalian cells depend on the presence in the cell membrane of active Na^+^,K^+^-ATPase creating and maintaining the indispensable Na^+^ and K^+^ concentration gradients. To study the ability of Na^+^,K^+^-ATPase mutants to support cell growth, we applied ouabain selection pressure [27,30] on transfected cells. When cDNA encoding an exogenous active Na^+^,K^+^-ATPase insensitive to the inhibitor ouabain is transfected into mammalian cells (COS-1 cells were used here), which endogenously express a Na^+^,K^+^-ATPase sensitive to ouabain, the expressed exogenous Na^+^,K^+^-ATPase will confer ouabain resistance to the cells, thus allowing them to survive and grow in the presence of ouabain, with the exogenous cDNA becoming stably integrated in the COS-1 cell genome. In contrast, cells expressing only the endogenous ouabain-sensitive Na^+^,K^+^-ATPase will die in the presence of ouabain, because cell survival depends on the presence of an active Na^+^,K^+^-ATPase. The rat α1 isoform is well known to be ouabain-insensitive, i.e., its ouabain affinity is >100-fold lower than that of the human isoform. Hence, we used cDNA encoding rat α1 in the present study, as well as cDNA encoding human α3 rendered ouabain-insensitive by introducing the mutations Q108R and N119D in the ouabain-binding site (“RDα3 wild type”), i.e., the same residues as present at these positions in rat α1 [27]. As expected, the rat α1 wild type and the RDα3 wild type were able to confer ouabain resistance to the COS-1 cells. On the other hand, introduction of the disease mutations α3-G358V and α3-I363N into the RDα3 resulted in proteins that in repeated transfections were unable to support cell growth in the presence of ouabain. The same was the case for the equivalent mutations, G363V and I368N, introduced in rat α1, indicating very low Na^+^ and K^+^ transport activity. Based on our previous experience with a variety of Na^+^,K^+^-ATPase mutants, the lack of ability to support cell growth indicates that the mutant exhibits less than 5% of the transport activity of the wild type [17]. By contrast, we found that rat α1 with alanine replacement of G363 (G363A) was compatible with survival and sustained growth of the COS-1 cells in the presence of ouabain, allowing the cells to form colonies and be expanded into stable cell lines. Plasma membranes were isolated from these cells, and the function of the G363A mutant relative to wild-type rat α1 was characterized in detail, as described below.

### 3.2. Oligomycin Effect and Expression Level

Functional studies were performed on plasma membranes isolated from the COS-1 cells expressing the G363A mutant or wild type. The membranes were permeabilized to allow access of the substrates, Na^+^, K^+^, and ATP, as well as Mg^2+^, from both sides of the membrane. The presence of 10 µM ouabain in the media ensured that the endogenous COS-1 cell Na^+^,K^+^-ATPase was silenced.

**Figure 2:**
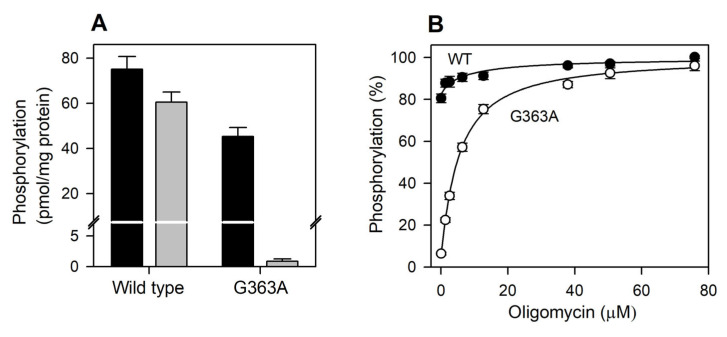
Na^+^-activated phosphorylation and dependence on oligomycin concentration. Experiments were performed with leaky plasma membrane preparations containing the wild type (left columns and filled circles) or the G363A mutant (right columns and open circles). Phosphorylation was performed for 10 s at 0 °C in the presence of 2 µM [γ-^32^P]ATP, 150 mM Na^+^ and the indicated concentrations of oligomycin. For further details, see Section 2.3. (**A**) Phosphorylation with (black columns) or without (gray columns) 25 µM oligomycin present. The ordinate shows pmol phosphorylation of the expressed exogenous wild type or G363A mutant per mg of total membrane protein. (**B**) Results of similar phosphorylation experiments conducted at a series of oligomycin concentrations. The ordinate shows the phosphorylation in % of the extrapolated value corresponding to infinite oligomycin concentration. Symbols with error bars (seen only when larger than the size of the symbols) represent the average of 10 or 11 independent experiments ± SEM. Each line illustrates the best fit of a Hill function (Equation (1) of Section 2.6). The extracted *A*_0_ (phosphorylation in the absence of oligomycin), *K*_0.5_ for oligomycin binding, and Hill coefficient were 82%, 6.9 ± 4.8 µM, and *n* = 0.9 for the wild type, and 6%, 5.4 ± 0.5 µM, and *n* = 1.1 for G363A.

First, we studied the effect of the antibiotic oligomycin on the Na^+^-activated phosphorylation from [γ-^32^P]ATP (Scheme 1, reaction 3). Oligomycin, best known as an inhibitor of the mitochondrial ATP synthase [35], also binds to the Na^+^,K^+^-ATPase at a site in the N-terminal region [6], which stabilizes the Na^+^-occluded state, thereby accelerating the phosphorylation reaction and slowing the Na^+^-releasing conversion of the [Na_3_]E_1_P form to E_2_P (reaction 4 in Scheme 1) [2,36,37]. The results in Figure 2A were obtained by performing the phosphorylation in the presence and absence of 25 µM oligomycin under conditions otherwise promotive of accumulation of the phosphoenzyme in the wild type, i.e., for 10 s at 0 °C in the presence of a saturating Na^+^ concentration of 150 mM and in the absence of K^+^. The absence of K^+^ ensures that the dephosphorylation of E_2_P (reactions 5 and 6 in Scheme 1) is relatively slow [2]. With 25 µM oligomycin present, the phosphorylation level determined for the wild type was 75 pmol/mg of total membrane protein, whereas in the absence of oligomycin it was 61 pmol/mg. For the G363A mutant, the phosphorylation level was almost negligible in the absence of oligomycin, but with oligomycin it increased to 45 pmol/mg (Figure 2A). The large difference between the phosphorylation levels of the mutant in the presence and absence of oligomycin indicates that the G363A mutation severely destabilizes the Na^+^-occluded state in the absence of oligomycin as stabilizer. The complete oligomycin concentration dependence of phosphoenzyme accumulation is shown in Figure 2B. The apparent affinities for oligomycin are almost equal for the wild type and the G363A mutant (*K*_0.5_ values 6.9 µM and 5.4 µM, respectively). The maximum phosphorylation levels corresponding to 100% oligomycin binding are 78 and 53 pmol/mg total membrane protein for the wild type and the G363A mutant, respectively, with the maximum level of the G363A mutant constituting 68% that of the wild type. The maximum phosphorylation level (“active site concentration” [33]) reflects the expression level of active enzyme. Hence, the G363A mutant is well expressed at a level comparable to that of the wild type, which allowed us to determine the effects of the mutation on Na^+^,K^+^-ATPase overall and partial reactions (Scheme 1) by steady-state and transient kinetic measurements.

### 3.3. Na^+^ Dependence of Phosphorylation

**Figure 3:**
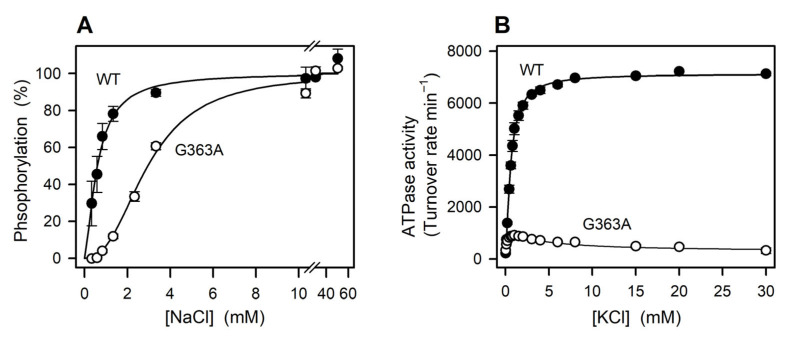
Na^+^ activation of phosphorylation and K^+^ concentration dependence of ATPase activity (catalytic turnover rate). Experiments were performed with leaky plasma membrane preparations containing the wild type (filled circles) or the G363A mutant (open circles). Symbols with error bars (seen only when larger than the size of the symbols) represent the average of 3 or 4 independent experiments ± SEM. (**A**) Na^+^ dependence of phosphorylation performed for 10 s at 0 °C in the presence of 2 µM [γ-^32^P]ATP, 25 µM oligomycin, and the indicated concentrations of Na^+^. For further details, see Section 2.3. The phosphorylation is shown in % of the maximal value (given in pmol/mg protein in Figure 2A, the black columns). Each line illustrates the best fit of a Hill function (Equation (1) of Section 2.6). The extracted *K*_0.5_ values for Na^+^ activation and Hill coefficient are 0.60 ± 0.07 mM and *n* = 1.5 for wild type, and 2.95 ± 0.09 mM and *n* = 2.4 for G363A. (**B**) ATPase activity measurements carried out at 37 °C in the presence of the indicated concentrations of K^+^ as well as 40 mM Na^+^ and 3 mM ATP. To obtain the catalytic turnover rate, the ATPase activity was divided by the active site concentration, defined as the maximal phosphorylation level corresponding to 100% oligomycin binding in Figure 2B. For further details, see Section 2.2. The lines illustrate the best fits of a Hill function without (wild type) or with (mutant G363A) a negative term added to represent inhibition (Equations (1) and (2), respectively, of Section 2.6).

The apparent affinity for Na^+^ binding at the three cytoplasmic-facing sites of E_1_ was studied by taking advantage of the requirement for binding of Na^+^ to these sites to activate the phosphoryl transfer from ATP (reactions 2 and 3 in Scheme 1). The phosphorylation with [γ-^32^P]ATP was determined at varying Na^+^ concentrations at 0 °C in the presence of oligomycin and absence of K^+^ to ensure accumulation of the maximal amount of phosphoenzyme (Figure 3A). The absence of K^+^ slows dephosphorylation (reactions 5 and 6 in Scheme 1), and furthermore prevents competition between Na^+^ and K^+^ at the cytoplasmic-facing sites (reactions 1 and 2 in Scheme 1), thereby avoiding the possibility that the apparent affinity determined for Na^+^ depends on the K^+^ affinity at these sites. The half-saturation constant *K*_0.5_ for Na^+^ binding extracted from the dependence of phosphorylation on Na^+^ concentration was 0.60 mM for the wild type and 2.95 mM for the G363A mutant. Hence, the apparent Na^+^ affinity (reciprocal of *K*_0.5_ for Na^+^) of the G363A mutant was fivefold reduced relative to that of the wild type.

### 3.4. K^+^ Concentration Dependence of Na^+^,K^+^-ATPase Activity and Catalytic Turnover Rate

Figure 3B shows the K^+^ concentration dependence of the Na^+^,K^+^-ATPase activity determined in the presence of 40 mM Na^+^ and optimal conditions with respect to ATP concentration and temperature (37 °C). The activity is expressed as the catalytic turnover rate in min^−1^, i.e., the ATP hydrolysis rate normalized to the expression level by taking the ratio between the ATP hydrolysis rate and the active site concentration determined by the phosphorylation corresponding to 100% oligomycin binding as described in Section 3.2. The maximal catalytic turnover rate of the G363A mutant is about eightfold lower than that of the wild type (911 min^−1^ versus 7215 min^−1^). K^+^ in the sub-millimolar concentration range stimulates the Na^+^,K^+^-ATPase activity in both the mutant and the wild type, due to the binding of K^+^ at the extracellular-facing sites of the K^+^-sensitive E_2_P conformation, which promotes dephosphorylation of the enzyme (reactions 5 and 6 in Scheme 1). However, the G363A mutant exhibits its maximal ATPase activity at a rather low K^+^ concentration of 1 mM, above which K^+^ is seen to inhibit the activity of the mutant (Equation (2) in Section 2.6). Such an inhibition phase is not seen for the wild-type enzyme under the prevailing conditions, but can be observed also for the wild type if the ratio between the Na^+^ and K^+^ concentrations is decreased further, either by reducing the concentration of Na^+^ or by increasing the K^+^ concentration [1]. The inhibition is caused by the binding of K^+^ at the cytoplasmic-facing E_1_ sites in competition with Na^+^. Hence, mutation G363A seems to increase the ability of K^+^ to compete with Na^+^ here, in accordance with the reduced affinity for Na^+^ binding at these sites observed in Figure 3A.

### 3.5. Rapid Kinetic Measurements of the Rate of Phosphorylation from ATP

**Figure 4:**
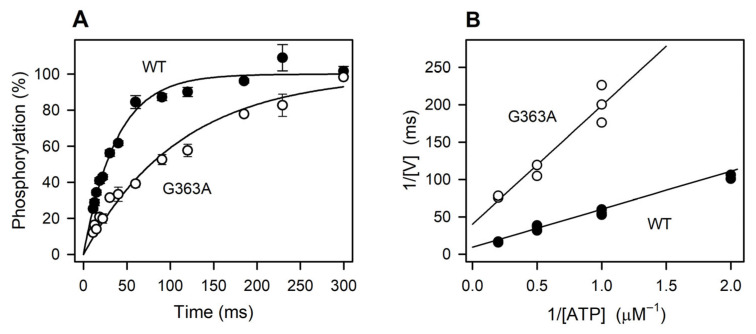
Rapid kinetic measurements of the phosphorylation rate and ATP concentration dependence. Experiments were performed with leaky plasma membrane preparations containing the wild type (filled circles) or the G363A mutant (open circles). A QFM-5 quench-flow module was used to follow the phosphorylation from [γ-^32^P]ATP at 25 °C in the presence of 100 mM Na^+^ and 25 µM oligomycin. For further details, see Section 2.3. (**A**) Phosphorylation time course at 2 µM [γ-^32^P]ATP. Symbols with error bars (seen only when larger than the size of the symbols) represent the average of two independent measurements ± SEM. Each line illustrates the best fit of a mono-exponential time function (Equation (3) of Section 2.6). The extracted rate constants are 27 s^−1^ and 9 s^−1^ for the wild type and the G363A mutant, respectively. (**B**) ATP concentration dependence of the initial rate of phosphorylation. The time course of phosphorylation was determined as described above in the presence of 0.5, 1, 2, or 5 µM [γ-^32^P]ATP. The graph shows a plot of the reciprocal of the initial rate (expressed relative to the enzyme concentration to obtain the molecular rate in ms^−1^) against the reciprocal of the ATP concentration (double reciprocal Lineweaver-Burk plot). Each data point represents one out of the 2–4 independent experiments performed for each ATP concentration. The lines illustrate the best fits of a linear function (representing the reciprocal of a Hill function (Equation (1) of Section 2.6) with *n* = 1 and *A*_0_ = 0. The maximal rate of the phosphorylation reaction corresponding to infinite ATP concentration and the *K*_0.5_ value for ATP (also known as the Michaelis constant, *K*_M_) extracted from these plots are 107 s^−1^ and 5.5 µM for wild type, and 25 s^−1^ and 3.9 µM for G363A.

Using quenched-flow methodology to perform kinetic measurements on a millisecond time scale [34], the time course of phosphorylation of the Na^+^-bound E_1_ form with [γ-^32^P]ATP (reaction 3 in Scheme 1) was studied at 25 °C in the presence of a saturating Na^+^ concentration of 100 mM and 25 µM oligomycin in the absence of K^+^. Figure 4A shows the phosphorylation time course for an ATP concentration of 2 µM. A mono-exponential time function (Equation (3) in Section 2.6) was fitted to the data, and the derived rate constant is threefold lower in the G363A mutant relative to that of the wild type, suggesting that either the phosphorylation reaction or the preceding binding of ATP is affected by the mutation. To distinguish between these possibilities, we determined the time course of phosphorylation at various ATP concentrations under conditions otherwise identical to those described for Figure 4A. The results in the form of a double reciprocal Lineweaver–Burk plot of the initial rate of phosphorylation as a function of the ATP concentration are shown in Figure 4B. The maximal phosphorylation rate is reduced about fourfold in the mutant relative to that of the wild type (25 s^−1^ versus 107 s^−1^), whereas the apparent affinity for ATP binding is not reduced in the mutant (*K*_0.5_ for the mutant 3.9 µM versus 5.5 µM for the wild type), thus indicating that it is the Na^+^-activated phosphoryl transfer reaction and not the binding of ATP to Na_3_E_1_ that is defective in the G363A mutant.

### 3.6. ATP Dependence of Na^+^,K^+^-ATPase Activity

**Figure 5:**
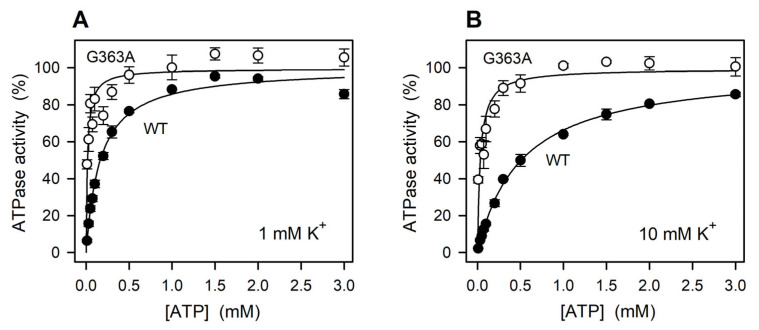
ATP concentration dependence of Na^+^,K^+^-ATPase activity at two K^+^ concentrations. Experiments were performed with leaky plasma membrane preparations containing the wild type (filled circles) or the G363A mutant (open circles). Symbols with error bars (seen only when larger than the size of the symbols) represent the average of 2–4 independent experiments ± SEM. ATPase activity measurements were carried out at 37 °C in the presence of the indicated concentrations of ATP as well as 130 mM Na^+^ and 1 mM K^+^ (**A**) or 10 mM K^+^ (**B**). For further details, see Section 2.2. Each line illustrates the best fit of a Hill function (Equation (1) of Section 2.6) with *A*_0_ = 0 and *n* = 1. The activity is shown in % of the extrapolated value corresponding to infinite ATP concentration. The extracted *K*_0.5_ values are for 1 mM K^+^ 165 ± 15 µM (wild type) and 17 ± 3 µM (G363A), and for 10 mM K^+^ 508 ± 48 µM (wild type) and 34 ± 5 µM (G363A).

The effect of ATP concentration on Na^+^,K^+^-ATPase activity at 37 °C was studied at K^+^ concentrations of 1 mM (Figure 5A) or 10 mM (Figure 5B), selected based on the results presented above in Figure 3B to represent conditions where mutant G363A either exhibits near-maximal Na^+^,K^+^-ATPase activity (1 mM K^+^) or is noticeably inhibited by K^+^ (10 mM K^+^). Relative to the wild type, G363A displays a conspicuous increase in the apparent ATP affinity at both K^+^ concentrations (*K*_0.5_ reduced 10-fold for 1 mM K^+^ and 15-fold for 10 mM K^+^). This behavior contrasts with the normal apparent affinity for ATP in the phosphorylation of Na_3_E_1_ described above in Section 3.5, but may be explained by a shift in the rate-limiting step of the overall Na^+^,K^+^-ATPase reaction caused by the mutation. It is well known that ATP binds with high affinity to the Na_3_E_1_ form (*K*_0.5_ in the micromolar range, as observed in Figure 4B), leading to phosphorylation, but binds with low affinity (*K*_0.5_ in the millimolar range) to the K^+^-occluded [K_2_]E_2_ form in a non-phosphorylating allosteric mode, accelerating the K^+^-deoccluding [K_2_]E_2_-to-E_1_ conversion (boxed ATP in reaction 1 of Scheme 1) [2,38]. Because the latter step is rate-limiting for the overall Na^+^,K^+^-ATPase reaction cycle of the wild-type enzyme, the apparent ATP affinity exhibited by the wild type in measurements of Na^+^,K^+^-ATPase activity is rather low, *K*_0.5_ in the 0.1–1 mM range. A mutation that shifts the rate-limiting step away from the [K_2_]E_2_-to-E_1_ conversion toward the phosphorylation of Na_3_E_1_ will therefore increase the apparent ATP affinity determined in measurements of Na^+^,K^+^-ATPase activity, as observed. The same dependence of the apparent ATP affinity on the accumulation of the K^+^-occluded [K_2_]E_2_ form also explains that the apparent ATP affinity is lower for 10 mM K^+^ than for 1 mM K^+^ in both the wild type and the G363A mutant.

### 3.7. Occlusion and Deocclusion of K^+^ and the K^+^ Congener Rb^+^

**Figure 6:**
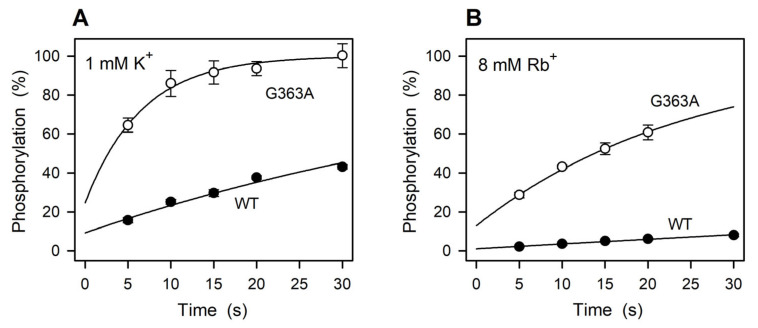
Time course of K^+^ and Rb^+^ deocclusion monitored by phosphorylation. Experiments were performed with leaky plasma membrane preparations containing the wild type (filled circles) or the G363A mutant (open circles). Symbols with error bars (seen only when larger than the size of the symbols) represent the average of 3–8 independent experiments ± SEM. Deocclusion and phosphorylation with [γ-^32^P]ATP was initiated by dilution of the K^+^- or Rb^+^-occluded intermediate formed by 1-h incubation with 1 mM K^+^ (**A**) or 8 mM Rb^+^ (**B**) into phosphorylation medium containing Na^+^ and [γ-^32^P]ATP. For further details, see Section 2.4. Phosphorylation is shown in % of the level with fully deoccluded enzyme obtained by performing the preincubation in the presence of 50 mM Na^+^ in place of K^+^ or Rb^+^. Each line illustrates the slow phase of a biphasic time function (Equation (4) in Section 2.6) fitted to the data. The ordinate intercept corresponding to the amplitude of the fast phase reflects the non-occluded E_1_ enzyme pool ready to bind Na^+^ and phosphorylate instantaneously, whereas the slow phase (rate constant *k*) reflects deocclusion of K^+^ or Rb^+^, its amplitude (100% minus the ordinate intercept) corresponding to the fraction of enzyme initially present in the occluded state (*E*_occluded_). The extracted values for *E*_occluded_ and *k* are for K^+^ 90.8 ± 1.6% and 0.017 ± 0.001 s^−1^ (wild type) and 75.4 ± 20.6% and 0.153 ± 0.045 s^−1^ (G363A), and for Rb^+^ 99.0 ± 0.4% and 0.0025 ± 0.0003 s^−1^ (wild type) and 87.1 ± 3.3% and 0.040 ± 0.004 s^−1^ (G363A).

Figure 6A shows the results of experiments in which the properties of the K^+^-occluded [K_2_]E_2_ enzyme were studied. Following equilibration with K^+^ to form [K_2_]E_2_ by the direct route [2], i.e., reversal of step 1 in Scheme 1, in the absence of Na^+^, the relative level of [K_2_]E_2_ as well as the rate of the K^+^-deoccluding [K_2_]E_2_-to-E_1_ conversion was determined. In the strategy used, the phosphorylation from [γ-^32^P]ATP is followed through the steps [K_2_]E_2_ ⟶ E_1_ ⟶ Na_3_E_1_ ⟶ [Na_3_]E_1_P (steps 1–3 in Scheme 1) under conditions where the deocclusion (step 1) is rate-limiting for the phosphorylation (temperature lowered to 10 °C). The phosphorylation reaction is initiated by a 10-fold dilution of the K^+^-equilibrated enzyme in a solution containing Na^+^ and [γ-^32^P]ATP as well as oligomycin, the latter being added just prior to phosphorylation to trap the phosphorylated enzyme. The data were analyzed by fitting a biphasic time function (Equation (4) in Section 2.6) in which the ordinate intercept corresponding to the fast phase reflects the non-occluded E_1_ enzyme pool ready to bind Na^+^ and phosphorylate almost instantaneously, whereas the exponential part (shown by the line) corresponds to the relatively slow deocclusion of K^+^ from [K_2_]E_2_ [33,34]. The fraction of the enzyme initially present as [K_2_]E_2_ equals 100% minus the ordinate intercept. The presence of both the rapid phase and the slow phase for the G363A mutant proves that the E_1_ ⟶ Na_3_E_1_ ⟶ [Na_3_]E_1_P part of the reaction sequence has not become rate-limiting for the observed phosphorylation of the mutant, even though Na_3_E_1_ ⟶ [Na_3_]E_1_P is slower in the mutant compared with the wild type, as demonstrated above in Figure 4A. On the contrary, the results in Figure 6A show that because of a faster deocclusion of K^+^, G363A phosphorylates considerably faster than the wild type when starting from the occluded form. The data show that G363A differs significantly from the wild type with respect to both the level of the occluded intermediate initially present (75% [K_2_]E_2_ vs. 91% for wild type) and the deocclusion rate (ninefold enhanced relative to wild type).

Similar experiments were conducted with the K^+^ congener Rb^+^, which due to its high affinity allowed most of the enzyme to reside in the occluded state upon equilibration with 8 mM Rb^+^ (Figure 6B). Hence, under these conditions, the level of the occluded intermediate initially present increased both for mutant G363A and for the wild type (87% [Rb_2_]E_2_ for the mutant versus 99% for wild type). Although the rate of deocclusion of Rb^+^ was lower than that of K^+^, it was 16-fold enhanced in the mutant relative to wild type. Hence, the data indicate that both [K_2_]E_2_ and [Rb_2_]E_2_ are destabilized in the mutant relative to wild type.

### 3.8. Effect of Rb^+^ on Affinity for Vanadate

**Figure 7:**
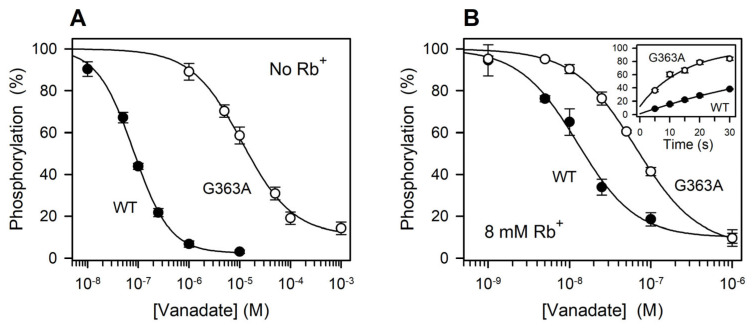
Affinity for vanadate in the absence and presence of Rb^+^. Experiments were performed with leaky plasma membrane preparations containing the wild type (filled circles) or the G363A mutant (open circles). Symbols with error bars (seen only when larger than the size of the symbols) represent the average of 4 independent experiments ± SEM. The enzyme was equilibrated with the indicated concentrations of orthovanadate in the absence (**A**) or presence (**B**) of 8 mM Rb^+^. The vanadate-free enzyme fraction was then determined by measuring the amount of phosphoenzyme formed upon dilution into phosphorylation medium containing Na^+^ and [γ-^32^P]ATP. For further details, see Section 2.5. The phosphorylation is shown in % of the maximal phosphorylation corresponding to a zero-vanadate concentration. Each line illustrates the best fit of a negative Hill function representing ligand-induced inhibition (Equation (5) in Section 2.6). The extracted *K*_0.5_ values for vanadate inhibition are in the absence of Rb^+^ 0.081 ± 0.009 µM (wild type) and 11.176 ± 1.367 µM (G363A), and in the presence of 8 mM Rb^+^ 0.013 ± 0.004 µM (wild type) and 0.066 ± 0.007 µM (G363A). The insert of Figure 7B shows the result of an experiment corresponding to that in Figure 6B (ordinate scale % phosphorylation), but conducted under medium and temperature conditions identical to those applied in the vanadate experiments, except for the absence of vanadate (for further details, see Section 2.4). Although in the insert of Figure 7B, the deocclusion rate is higher than in Figure 6B, due to the higher concentration of ATP that accelerates deocclusion (cf the boxed ATP in Scheme 1, reaction 1), the fraction of the enzyme initially present in the Rb^+^-occluded state (*E*_occluded_) is 99.7% for wild type and 87.6% for G363A, i.e., rather similar to that determined in Figure 6B, showing that most of the enzyme was trapped in the in the [Rb_2_]E_2_ form during vanadate binding.

Vanadate (orthovanadate) is an analog of phosphate in the [K_2_]E_2_·P transition state complex from which inorganic phosphate is released during forward cycling of the enzyme [39] (cf Scheme 1, step 6). Hence, vanadate inhibits Na^+^,K^+^-ATPase activity by binding with high affinity in competition with P_i_ to the phosphorylation site of the E_2_ and [K_2_]E_2_ or [Rb_2_]E_2_ conformations, thereby locking the enzyme in a stable dead-end state. Because vanadate binds specifically to the E_2_ conformations of the dephosphoenzyme, but not to E_1_, determination of the affinity of G363A for vanadate provides information about the effect of the mutation on the equilibrium between E_1_ and E_2_. The wild-type or mutant Na^+^,K^+^-ATPase is first equilibrated with various concentrations of vanadate in the absence of Na^+^ and ATP. Then, the vanadate-free enzyme fraction, which corresponds to the E_1_ enzyme pool, is determined as the fraction of enzyme able to form a phosphoenzyme with [γ-^32^P]ATP at 0 °C. The phosphorylation level obtained in this way reflects the equilibrium between the vanadate-free (E_1_ form) and the vanadate-bound enzyme (E_2_ forms) existing before initiation of phosphorylation. This assay was conducted both in the absence and presence of Rb^+^ (Figure 7A and Figure 7B, respectively). The latter condition ensured that most of the enzyme was trapped in the [Rb_2_]E_2_ form, as revealed in the measurements of occlusion and deocclusion of Rb^+^ shown in Figure 6B and the insert of Figure 7B (in the latter, the measurements were conducted under medium and temperature conditions identical to those applied in the vanadate experiments except for the absence of vanadate, which did not affect the ordinate intercept appreciably compared with Figure 6B). Accumulation of the [Rb_2_]E_2_ intermediate eliminates the effect of the G363A mutation on the E_1_–E_2_ conformational equilibrium. Hence, in the absence of Rb^+^, G363A displays a remarkable 138-fold reduction in the apparent vanadate affinity relative to the wild type. In the presence of Rb^+^, however, G363A only displays a fivefold-reduced affinity for vanadate relative to the wild type, indicating that a major part of the reduction of vanadate affinity seen for the mutant in the absence of Rb^+^ is due to a shift in the E_2_−E_1_ conformational equilibrium in favor of E_1_, rather than a reduced intrinsic affinity of E_2_ for vanadate, relative to wild type.

## 4. Discussion

The highly conserved glycine in focus in this study and the nearby isoleucine of the Na^+^,K^+^-ATPase α3 isoform are targets of mutations (α3-G358V and α3-I363N) associated with some of the most severe neurological phenotypes known for α3 mutations [24]. Accordingly, we found these disease mutations incompatible with survival and growth of transfected cells, when introduced either in human α3 or in rat α1 Na^+^,K^+^-ATPase. Because a previous study showed that α3-G358V and α3-I363N are well expressed in the plasma membrane [24], it can be concluded that the function of the Na^+^,K^+^-ATPase, and not the biosynthesis, is severely impaired by these mutations. More detailed information about the functional role of the glycine was obtained by introducing the alanine mutation of the glycine in the rat α1 Na^+^,K^+^-ATPase (G363A). This mutant is also well expressed and supports cell growth, thus indicating that a certain level of Na^+^,K^+^-transport activity is retained with the G363A mutation. Hence, the function of the mutant was able to be analyzed in detail on isolated plasma membranes.

The E_1_ form of the wild-type Na^+^,K^+^-ATPase binds three Na^+^ with high affinity at cytoplasmic-facing binding sites, denoted sites I, II, and III (Figure 1 and reaction 2 in Scheme 1). Na^+^ binding is sequential and cooperative, site III being occupied first and site II last, triggering the phosphoryl transfer from ATP [6] with occlusion of the three bound Na^+^ ions (reaction 3 in Scheme 1), i.e., the bound Na^+^ ions no longer have access to the medium on either side of the membrane due to the positioning of certain hydrophobic residues [7]. The E_2_ form, on the other hand, is unable to become phosphorylated from ATP and contains two K^+^ in an occluded state at sites formed by almost the same amino-acid side chains that constitute Na^+^ sites I and II. The two K^+^ enter the occluded state in [K_2_]E_2_ by binding either with high affinity from the extracellular side to the E_2_P form (reaction 5 in Scheme 1) or by binding with low affinity from the cytoplasmic side to E_1_ in competition with Na^+^ (reaction 1 in Scheme 1, the so-called direct route of K^+^ occlusion [2]).

Our results show that the Na^+^-activated phosphorylation from ATP is defective in mutant G363A, which explains the strongly reduced catalytic turnover rate (Na^+^,K^+^-ATPase activity per active site, Figure 3B). First, the steady-state phosphorylation level of mutant G363A is unusually low in the absence of oligomycin compared with that determined in the presence of oligomycin (Figure 2A,B). The difference between the phosphorylation levels of the mutant in the presence and absence of oligomycin is even larger than the conspicuous difference previously reported [33] for a Na^+^,K^+^-ATPase mutant with alteration to the Na^+^-binding glutamate in transmembrane segment M4 (E329 in Figure 1), a residue known to play a key role in Na^+^ occlusion and signal transduction to the phosphorylation site [6,7,33]. Because oligomycin promotes Na^+^ occlusion [36,37] by stabilizing a particular configuration of the M1–M4 helices [6], thereby allowing the phosphorylated [Na_3_]E_1_P state to accumulate, it is likely that the low steady-state phosphorylation level observed for the G363A mutant in the absence of oligomycin is caused by defective Na^+^ occlusion. Second, even in the presence of oligomycin, the Na^+^ affinity for activation of phosphorylation from ATP is reduced fivefold in G363A (Figure 3A). A similar experiment could not be conducted in the absence of oligomycin due to the very low amount of phosphoenzyme accumulated under these conditions. However, a reduced Na^+^ affinity of the E_1_ form of G363A in the absence of oligomycin can be deduced from the inhibition of ATPase activity in G363A by relatively low concentrations of K^+^, reflecting an enhanced ability of K^+^ to compete with Na^+^ at the cytoplasmic-facing binding sites of E_1_ (Figure 3B). Third, the maximal phosphorylation rate was found reduced by fourfold in G363A relative to the wild type without reduction in ATP affinity (Figure 4). These data all point to a defect in Na^+^ occlusion and its coupling with phosphoryl transfer from ATP.

Another major defect in the function of the G363A mutant relates to the E_2_−E_1_ conformational transition and the occlusion of K^+^ and its congener Rb^+^ in the E_2_ form. We found that the amount of K^+^- or Rb^+^-occluded enzyme, [K_2_]E_2_/[Rb_2_]E_2_, formed at equilibrium by the direct route (reaction 1 in Scheme 1) is reduced in the G363A mutant relative to the wild type and that the rate of deocclusion is much higher in the mutant than in the wild type (9- and 16-fold enhanced for K^+^ and Rb^+^, respectively), indicating a destabilization of the K^+^- or Rb^+^-occluded E_2_ state in G363A (Figure 6). This result is in line with the ATP concentration dependence of steady-state Na^+^,K^+^-ATPase activity, showing an increased apparent ATP affinity of G363A relative to the wild type (Figure 5). ATP normally binds with low affinity to the K^+^-occluded [K_2_]E_2_ intermediate, opening the cytoplasmic headpiece and thereby accelerating the [K_2_]E_2_-to-E_1_ conversion with deocclusion of K^+^ [2,7,38]. A mutation that, like ATP binding, destabilizes [K_2_]E_2_ will therefore shift the ATP concentration dependence toward lower ATP concentrations, i.e., increase the apparent ATP affinity. Further evidence supporting that the G363A mutation displaces the E_2_−E_1_ conformational equilibrium in favor of E_1_ was obtained in the studies of vanadate binding (Figure 7). Because vanadate binds exclusively to E_2_ forms, the conspicuous reduction in apparent affinity for vanadate observed with the G363A mutant is in accordance with an E_1_-poise of the conformational equilibrium. A reduced intrinsic affinity of E_2_ for vanadate might also explain the data in Figure 7A. However, by taking advantage of Rb^+^ as a high-affinity congener of K^+^ that displaces the E_2_−E_1_ equilibrium almost entirely toward E_2_, even in the mutant, it was possible to demonstrate that a major part of the reduction of vanadate affinity seen in the absence of Rb^+^ is caused by a shift of the E_2_−E_1_ conformational equilibrium in favor of E_1_.

In principle, the displacement of the E_2_−E_1_ conformational equilibrium in favor of E_1_ in G363A described above would be expected to increase the apparent Na^+^ affinity for activation of phosphorylation from ATP, because E_1_ is the form able to bind Na^+^ with high affinity and phosphorylate. However, because the opposite was observed with the mutant, i.e., Na^+^ affinity being reduced, this effect on Na^+^ affinity must be intrinsic to the E_1_ form of the mutant. In fact, the reduction of Na^+^ affinity of E_1_ may be even more pronounced than the fivefold reduction revealed in the phosphorylation assay, because it may be partially masked by the displacement of the E_2_−E_1_ conformational equilibrium in favor of E_1_.

Hence, the functional measurements presented here suggest that the Na^+^- and K^+^-occluded states are destabilized by the G363A mutation. Further support of this hypothesis can possibly be obtained in future studies by determination of the structures of the Na^+^- and K^+^-bound states of the mutant by cryo-EM. To understand that alteration to G363 strongly affects Na^+^ occlusion and its coupling with phosphoryl transfer from ATP as well as the stability of the E_2_ form, it is important to remember that the glycine is connected via the β1 strand of the N-terminal part of the P domain to the phosphorylated aspartate of the catalytic site as well as via the P1 helix to transmembrane helix M4, which is critical to ion occlusion and translocation [6,7,33]. An essential difference between the E_1_ and E_2_ forms in the wild type is that the M4 helix, which contributes four of the five oxygen ligands of site II (V327 and E329 are shown on Figure 1), is displaced toward the cytoplasmic side in E_1_ relative to its position in E_2_. This allows Na^+^ to bind with high affinity, replacing K^+^ (cf Scheme 1). The binding of the last of the three Na^+^ ions to E_1_ at site II completes this movement by bringing the phosphorylation site aspartate in the P domain in the correct position to receive the γ-phosphate of ATP [7]. The flexibility imposed in the wild type by the presence of the small glycine, lacking a side chain, at the joining point between the P1 helix and β1 may well be critical for the rearrangement that moves M4 toward the cytoplasmic side and couples Na^+^ binding with phosphoryl transfer through changes involving the P1 helix and β1. This flexibility is lost with replacement of the glycine by alanine, having a side chain. The change in direction of the backbone chain imposed by the presence of a side chain at this position may in addition disrupt the hydrogen bond between the backbone carbonyl and the centrally located arginine in transmembrane helix M5 (cf Figure 1, R761 in rat α1, R756 in human α3). In the wild type, this arginine forms hydrogen bonds with the glycine as well as with the C-terminal part of the P domain that continues into M5 and with the cytoplasmic loop between transmembrane helices M6 and M7, thus promoting interaction between these functional subdomains (Figure 1 and [25]). A bend of the M5 helix present in the [K_2_]E_2_ form is straightened during the [K_2_]E_2_-to-E_1_ transition, which forces M4 toward the cytoplasmic side, because interaction between residues on the M4–M5 interface is prevented by the change in disposition of M5 [7]. Disruption of the hydrogen-bond interconnection between the M4–P1–β1 sector and M5 by the G363A mutation may add further to the destabilization of the [K_2_]E_2_ form caused by the straightening of M5, thereby promoting the [K_2_]E_2_-to-E_1_ transition (reaction 1 in Scheme 1), as observed. While this effect leads to a relative stabilization of the unphosphorylated E_1_ form with no Na^+^ or K^+^ bound, the disruption of the hydrogen bond between G363 and R761 in the mutant may in addition interfere with the final steps (2 and 3 in Scheme 1) in the rearrangement of the M4–P1–β1 sector that lead to occlusion of Na^+^ and bring the P domain aspartate in correct position for phosphoryl transfer.

The loss of function of the two neurological disease mutants α3-G358V and α3-I363N as well as of the rat α1 equivalents G363V and I368N can be understood along the same lines as described above for the rat α1-G363A, but with more fatal consequences in terms of impaired Na^+^ activation of phosphorylation. Hence, the bulkier valine should be much more disturbing than alanine for the disposition of the M4–P1–β1 connection to the phosphorylated aspartate (accessible side-chain volume of valine 117 Å^3^ versus 67 Å^3^ for alanine [40]). The α3-I363N/rat α1-I368N introduces a polar side chain in place of the bulky hydrophobic isoleucine in the β1 strand only three residues from the phosphorylated aspartate (rat α1-D371, see Figure 1), which will disturb the course of β1 that in the wild type is fixed by the backbone hydrogen bonds with two residues (K607 and I609 in Figure 1) in the parallel β2 strand of the P domain and by interaction of the isoleucine side chain with hydrophobic residues at the cytoplasmic end of M5.

## 5. Conclusions

While the disease mutations α3-G358V and α3-I363N affect Na^+^,K^+^-ATPase function to an extent where it is unable to support cell growth, indicating very low Na^+^ and K^+^ transport activity, the rat α1 mutant G363A supported cell growth, and although its function was strongly impaired, it was able to be characterized in detail in isolated plasma membranes. The eightfold reduction in Na^+^,K^+^-ATPase activity of G363A relative to wild type was traced to a reduced rate of the Na^+^-activated phosphorylation from ATP. Occlusion of Na^+^ appears to be defective in mutant G363A, resulting in lack of accumulation of the Na^+^-occluded phosphorylated intermediate in the absence of oligomycin to support Na^+^ occlusion, as well as in reduced Na^+^ affinity and reduced phosphorylation rate even in the presence of oligomycin. In addition, the G363A mutation displaces the E_2_−E_1_ conformational equilibrium in favor of E_1_, leading to destabilization of the K^+^-occluded state and a faster deocclusion of K^+^. Together, these effects of the G363A mutation must lead to accumulation of a non-productive E_1_ state with no Na^+^ or K^+^ bound. These functional changes can be understood based on the structural location of the glycine at a strategic position in the M4–P1–β1 sector, which connects the ion-binding region of transmembrane helix M4 with the catalytic ATP hydrolysis site in the P domain and with transmembrane helix M5. These regions need to be well coordinated in their movements coupling K^+^ deocclusion and Na^+^ occlusion with activation of phosphorylation.

Whether and how the haploinsufficiency resulting from loss of function of the neurological disease mutants can explain the severe neurological phenotypes is not clear at present [41] and requires further experimentation, possibly including in vivo studies. A perspective of the insight provided by the present study is that it might help development of efficient drugs to treat patients with neurological disease caused by mutations in Na^+^,K^+^-ATPase that affect Na^+^ occlusion. The goal should be to obtain a pharmaceutical that mimics the effect of oligomycin on Na^+^,K^+^-ATPase, but without its toxic effects on the mitochondrial ATP synthase.

## Data Availability

The original contributions presented in this study are included in the article. Further inquiries can be directed to the corresponding authors.
